# Pelvic organ prolapse after laparoscopic hysterectomy compared with vaginal hysterectomy: the POP-UP study

**DOI:** 10.1007/s00192-020-04591-z

**Published:** 2020-11-10

**Authors:** Carolien K. M. Vermeulen, Joggem Veen, Caroline Adang, Sanne A. L. van Leijsen, Anne-Lotte W. M. Coolen, Marlies Y. Bongers

**Affiliations:** 1 Department of Gynecology and Obstetrics, Máxima Medical Centre, De Run 4600, 5500 MB Veldhoven, The Netherlands; 2 Department of Gynecology and Obstetrics, Maastricht University Medical Center, P. Debyelaan 25, 6229 HX Maastricht, The Netherlands; 3 GROW, Research School of Oncology and Developmental Biology, University of Maastricht, Universiteitssingel 40, 6229 ER Maastricht, The Netherlands

**Keywords:** Pelvic organ prolapse, Hysterectomy, Vaginal hysterectomy, Laparoscopic hysterectomy, Epidemiology

## Abstract

**Introduction and hypothesis:**

The objective was to review the long-term prevalence of pelvic organ prolapse (POP) after laparoscopic hysterectomy (LH) compared with vaginal hysterectomy (VH).

**Methods:**

An observational cohort study was conducted amongst women who underwent an LH or a VH for benign indications during the period 1996–2004: the POP-UP study. The prevalence of POP was inventoried by a questionnaire involving the Pelvic Floor Distress Inventory (PFDI-20) and a pelvic floor examination (POP-Q). Women were divided into groups based on route and indication of hysterectomy: LH, VH-1 (for nonprolapse), and VH-2 (prolapse).

**Results:**

Four hundred and six of the 706 eligible patients (58%) returned the questionnaire and 247 underwent POP-Q examination. Sixty-eight patients (17%) received treatment for prolapse; 8% LH, 10% VH-1, and 29% VH-2 (Chi-squared test, *p* < 0.001). The prevalence of vaginal vault prolapse (apical surgery or ≥ stage 2 at POP-Q) was 4.4% for LH and 5.8% for VH-1 (*p* = 0.707); and 23% for VH-2 (VH-2 versus others, *p* < 0.0001). The prevalence of prolapse ≥ stage 2 in any compartment was 62% (*n* = 153) in total and in 42% of the LH group, 51% of the VH-1 group, and 84% of the VH-2 group (Chi-squared test, *p* < 0.001). A symptomatic POP (anatomical POP ≥ stage 2 with bulging) was present in 11% of the population.

**Conclusions:**

No difference was found in the prevalence of POP between LH and VH for nonprolapse indications. However, POP after VH for prolapse occurs more frequently than after hysterectomy for other indications.

## Introduction

Pelvic organ prolapse (POP) is a common condition that can cause serious discomfort and is often associated with feelings of uncertainty and shame [1]. Of women aged 45 years or older, 38.5% has an anatomical POP that may require surgical repair [2, 3]. A life-time risk for POP surgery of 11–20% was described in epidemiological studies, with an increasing societal burden due to a recurrence rate of 29% and an aging population [2, 4–6]. It is clear that the risk for POP should be minimized where possible.

Hysterectomy is a proven risk factor for POP, and also one of the top ten most common surgeries performed amongst women. Several studies confirm the relationship between vaginal hysterectomy (VH) and POP surgery, mostly occurring within 2–5 years after the hysterectomy [7, 8]. The median time interval from hysterectomy—of any kind—and POP surgery is 6.2 to 13.7 years in literature [7–10]. The hazard ratio was highest after VH with POP as indication [3, 9, 11, 12]. Uterine descent is necessary in order to perform VH and the surgery itself is hypothesized to contribute to pelvic floor weakness [8, 11]. Abdominal hysterectomy (AH) was also positively related to POP surgery, owing to inevitable damage of the supportive tissues [7, 8].

Laparoscopic surgery has gained popularity over time because of the advantage of favorable peri- and postoperative outcome compared with abdominal surgery [13–16]. The uterosacral ligaments are of major importance for the level one pelvic organ support [17], but it is unclear how the ligaments are affected by the laparoscopic hysterectomy. Hypothetically, the uterosacral ligaments are damaged less during laparoscopy than during the open abdominal and vaginal approaches. Whether this leads to a different prevalence of POP later in life is as yet unexplored.

Most epidemiological studies use POP surgery as outcome measure. Data of women with asymptomatic POP, symptomatic women who do not seek help, and women with conservatively treated POP are scarce. The true POP incidence may therefore be underestimated.

The objective of this study is to compare the prevalence of anatomical and symptomatic POP after laparoscopic compared with vaginal hysterectomy.

## Materials and methods

In 2017, a questionnaire and invitation for a vaginal examination were sent to women who underwent either laparoscopic or vaginal hysterectomy in a single-center teaching hospital during the period 1996 to 2004. The timeframe is based on the upper limit of the median time interval between hysterectomy and POP surgery found in the literature, going backward from there as far as possible to 1996 in order to secure an adequate follow-up period.

The research protocol was approved by the Medical Research Ethics Committee of the Máxima Medical Center (24th February 2017, NL60096.015.16). The study is registered in the Dutch Trial Registry: Trial NL5967 (NTR6333). All participants signed informed consent before inclusion. This study was developed and described in accordance with the strengthening the reporting of observational studies in epidemiology (STROBE) guidelines [18]. We named our study by the acronym the “POP-UP” study.

To identify eligible women, the surgical registry of the hospital was digitally accessed and a list of all hysterectomies (vaginal, laparoscopic, supracervical, and abdominal) performed between 1999 and 2004 was obtained. From this list, patients with vaginal and laparoscopic hysterectomy were selected. For the period 1996–1999, such a list was not available. Therefore, a list of all gynecological procedures was obtained and screened for vaginal and laparoscopic hysterectomies.

The laparoscopic hysterectomy was performed by two different gynecologists and the vaginal hysterectomy by four gynecologists. In both the vaginal and laparoscopic hysterectomies the vaginal vault was attached to the uterosacral ligaments with absorbable sutures. During laparoscopy, the vagina was opened cranially to the uterosacral ligaments, leaving the tissue where the ligaments come together intact.

Women were included if the hysterectomy was performed for a benign indication. Women were excluded if the cervix was left in place and if the hysterectomy was performed for malignant disease. Finally, women who were deceased or who were over 80 years old at the time of study participation were excluded, as the journey to the hospital for gynecological examination was considered too high a burden for women in this age group.

The primary outcome of this study is the prevalence of vaginal vault prolapse, which is defined as the total number of women who had POP surgery for vaginal vault prolapse after hysterectomy plus the women with anatomical POP of the apical compartment ≥ stage 2 found at gynecological examination. The secondary outcomes were defined as: the presence of prolapse ≥ stage 2 in other compartments, symptomatic POP (defined as bulging plus any ≥ stage 2 prolapse), pelvic floor symptoms and treatment for POP after hysterectomy. We will also present a commonly used composite outcome, based on the Barber criteria of success [19]: 1. prolapse beyond the hymen, or 2. bulging, or 3. a history of POP surgery.

Pelvic floor symptoms were inventoried by the Pelvic Floor Distress Inventory (PFDI-20), validated in Dutch [20], which can be divided into three subcategories: Pelvic Organ Prolapse Distress Inventory (POPDI-6), Colorectal-Anal Distress Inventory (CRADI-8), and Urinary Distress Inventory (UDI-6). The score quantifies complaints and bother over the past 3 months. A prolapse was called symptomatic if mild, moderate, or severe bulging was present.

Risk factors for POP such as age, BMI, profession and obstetric history [1, 8, 9, 11, 21] were part of the survey and analyzed as well. The patient charts (on paper or digital) of all participants were reviewed for type of hysterectomy, indication for hysterectomy, concomitant procedures (such as prolapse repairs) at hysterectomy and POP surgery (year, compartment of POP and type of procedure) in the follow-up time in order to reduce recall bias.

After 1 month, a reminder was sent to the nonresponders, who were contacted by phone to ask them to participate. If women decided not to participate, a short telephone interview was attempted in order to estimate response bias. Information gathered by phone included a history of prolapse treatment, micturition/defecation complaints, and vaginal bulging, as this is considered the most sensitive question for detecting a POP [22]. Women were lost to follow-up if the contact details appeared to be inaccurate (survey received “return to sender” and/or a disconnected telephone number).

The presence of POP was examined at the outpatient clinic by physicians authorized to perform the standardized Pelvic Organ Prolapse Quantification (POP-Q) [23]. The examiner was blinded to the type of hysterectomy, indication for hysterectomy, and other gynecological history. A prolapse of POP-Q stage 2 or higher (≥ −1 cm toward the hymen) was defined as an anatomical prolapse.

For accurate analysis, the women were divided into three groups. Women with a laparoscopic hysterectomy (LH), women with a vaginal hysterectomy for all benign indications except prolapse (VH-1), and women who were treated for POP by vaginal hysterectomy (VH-2). In this hospital laparoscopic hysterectomy was never performed for POP.

The data obtained from the surveys was entered manually into an SPSS database. To minimalize human error, the database was completely checked by a second person. Data analysis was performed using SPSS 22.0 (SPSS, Chicago, IL, USA). For continuous variables, results are expressed as median and range, and significance calculated using the independent *t* test. In the case of ordinal variables, proportions were calculated and the Chi-squared test was performed, or a Fisher’s exact test in the case of counts <5. A *p* value <0.05 was considered statistically significant. Confounding variables were identified amongst risk factors such as age, BMI, and obstetric history, and a univariate logistic regression analysis was performed to correct for these variables.

## Results

### Population

**Fig. 1:**
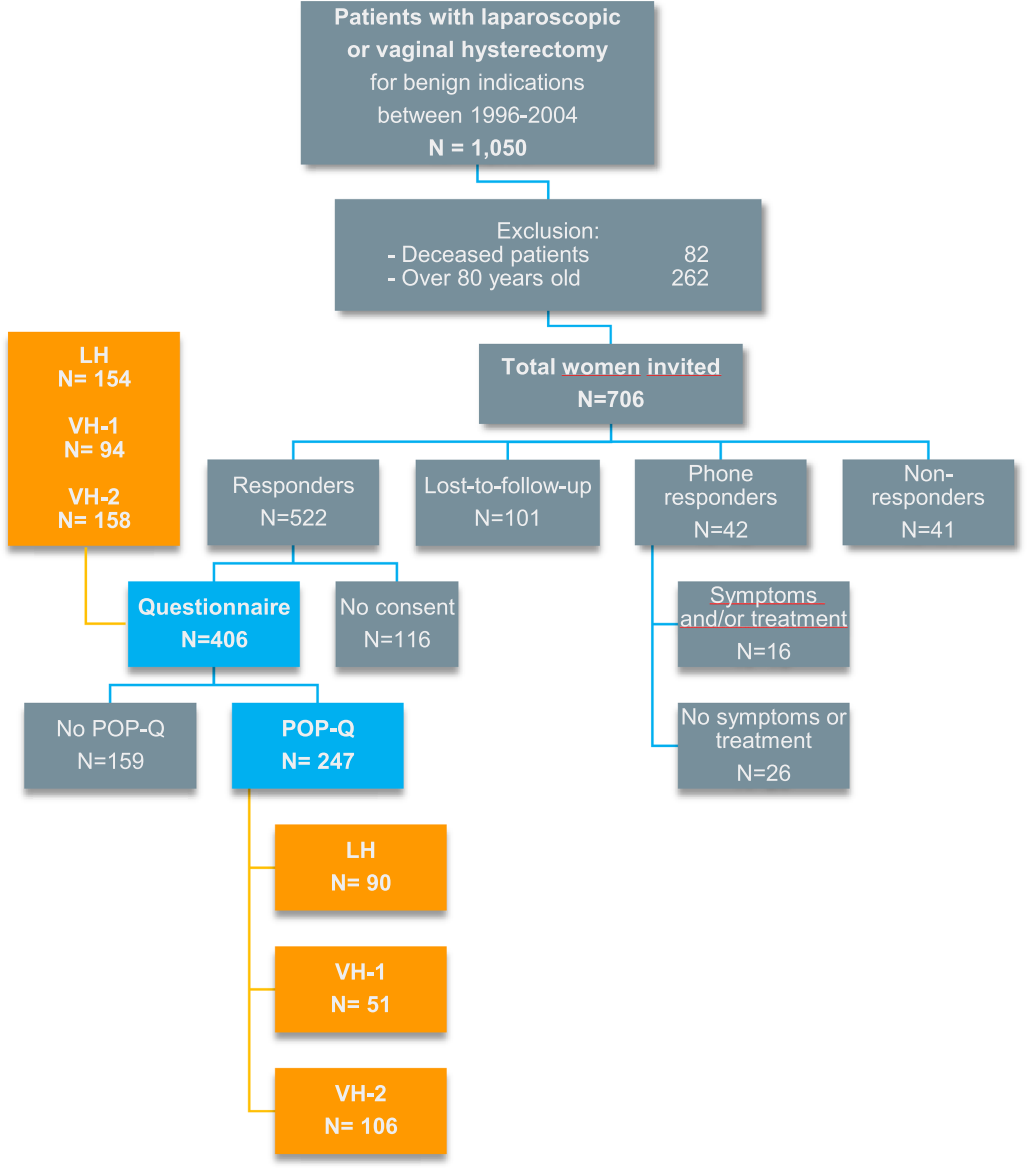
Study design and population. *LH* laparoscopic hysterectomy, *POP-Q* Pelvic Organ Prolapse Quantification, *VH* vaginal hysterectomy

From our hospital inpatient enquiry system we identified 1,050 women with a VH or LH for benign indications during the period 1996–2004. Eighty-two women were deceased and 262 women were over 80 years old and therefore excluded. The remaining 706 women were eligible, of whom 406 (58%) responded to the questionnaire by mail and 247 (35%) gave consent to POP-Q examination (Fig. 1). One hundred and one women were lost to follow-up. Eighty-three women did not respond to the invitation by post, but 42 of these women consented to answer the short telephone interview.

The LH group consisted of 154 women who filled out the questionnaire and 90 of them also agreed to be examined for POP. For the VH-1 group this was 94 and 51 women respectively, and for the VH-2 group this was 158 and 106 women respectively.

Of the 42 women who consented to the short telephone interview, 16 (38%) did have current symptoms or previous treatment for prolapse in the past and 26 (62%) had no symptoms and no history of prolapse treatment.

Figure 1 shows the population enrollment.

### Baseline characteristics

The baseline characteristics of the POP-Q population (all women who attended our outpatient clinic for POP-Q examination, *N* = 247) are presented in Table 1. Parity was the only statistically significant difference at baseline between the LH and VH-1 group (*p* = 0.020). VH-2 women were statistically significantly older (*p* < 0.001), had undergone more vaginal deliveries (*p* < 0.001), but fewer vaginally assisted deliveries (*p* = 0.030) than the LH/VH-1 group. Concomitant POP surgery at the time of hysterectomy occurred in almost all women of the VH-2 group and is therefore a significantly different baseline characteristic. All procedures were native tissue repairs; the particular compartments reconstructed are shown in Table 1.

**Table 1:** Baseline characteristics of the pelvic organ prolapse quantification (POP-Q) population

	LH (*n* = 90)	VH-1 (*n* = 51)	VH-2 (*n* = 106)	LH vs VH-1 *p* value*	VH-2 vs other *p* value*
Age, median and range (years)	62 (47–77)	61 (48–70)	68 (45–80)	0.262	< 0.001
Body mass index, median and range	25 (19–45)	26 (19–40)	26 (19–40)	0.860	0.669
Parity, *n* (%)					
No vaginal delivery	24 (27)	1 (2)	1 (1)	0.020	< 0.001
1 vaginal delivery	10 (11)	8 (16)	11 (10)		
2+ vaginal deliveries	56 (62)	42 (82)	94 (89)		
Concomitant POP surgery during hysterectomy	3 (3)	1 (2)	97 (92)	0.637	< 0.001
Combined anterior and posterior wall repair	0	0	76 (72)		
Anterior wall repair	0	1 (2)	17 (16)		
Posterior wall repair	3 (3)	0	4 (4)		
Assisted vaginal delivery, *n* (%)	14 (16)	8 (16)	7 (7)	0.984	0.030
Vacuum	8 (9)	2 (4)	5 (5)		
Forceps	6 (7)	6 (12)	2 (2)		
Children with birth weight > 4,000 g, *n* (%)	16 (18)	12 (24)	31 (29)	0.412	0.087
Physically demanding profession^1^, *n* (%)	3 (3)	5 (10)	5 (5)	0.138	0.964

*LH* laparoscopic hysterectomy, *VH* vaginal hysterectomy

*Chi-squared test

^a^Profession was categorized as physically demanding to the best judgment of the data analyst (e.g., nurse scored “physically demanding” and administrative worker scored as “not physically demanding”)

### Results of the questionnaire

In total, 406 women responded the questionnaire, of which 154 (38%) were in the LH group, 94 (23%) were in the VH-1 group, and 158 (39%) were in the VH-2 group.

### PFDI-20 results

**Fig. 2:**
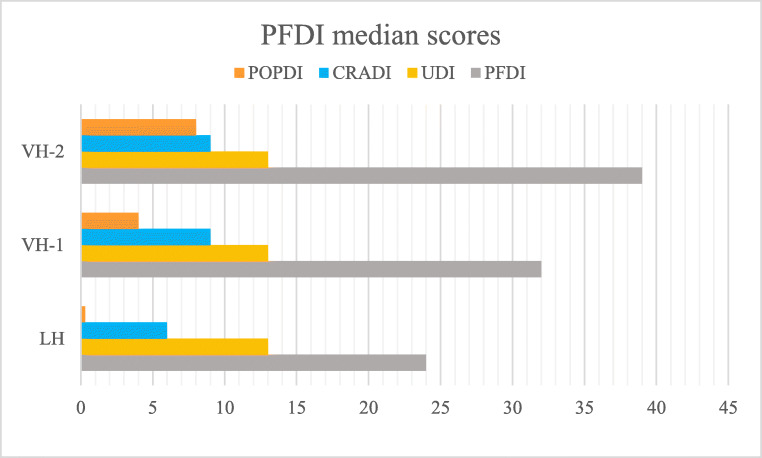
Median scores of the Pelvic Floor Distress Inventory (PFDI) questionnaire per patient group (*n* = 406). The PFDI score is the total of the Pelvic Organ Prolapse Distress Inventory (*POPDI*) score (bulging), Colorectal-Anal Distress Inventory (*CRADI*) score (defecation), and Urinary Distress Inventory (*UDI*) score (micturition). *LH* laparoscopic hysterectomy, *VH* vaginal hysterectomy

No statistically significant difference in overall pelvic floor complaints was found between LH and VH-1 (*p* = 0.884), but in comparison women in the VH-2 group perceived more pelvic floor symptoms (*p* = 0.013). A graphic overview is shown in Fig. 2.

Analyzing the three subcategories separately, the median score of the POPDI-6 was 0, 4, and 8 points for LH, VH-1, and VH-2 respectively, which was significantly different for VH-2 versus others (*p* = 0.005), but not for LH versus VH-1 (*p* = 0.532). The median scores of the CRADI-8 showed no significant differences between groups (VH-2 versus others, *p* = 0.076, and LH versus VH-1, *p* = 0.992). The median score of the UDI-6 was the same in all groups (LH, VH-1, and VH-2; Fig. 2).

Analysis of the PFDI-20 question about bulging “Do you usually have a bulge or something falling out that you can see or feel in your vaginal area?” showed mild, moderate or severe symptoms in 40 women (10%), of whom 9 were in the LH group (6%), 8 were in the VH-1 group (9%), and 23 women were in the VH-2 group (15%). Women in the VH-2 group report more bulging complaints than the others (*p* = 0.011). No difference was found between the LH and VH-1 groups.

### Prolapse treatment

According to the questionnaires (*N* = 406), 66 women (16%) were treated for prolapse following hysterectomy, either conservatively or surgically: in the LH group 9% (*n* = 14), VH-1 group 10% (*n* = 9), and VH-2 group 27% (*n* = 43), which was significantly more frequent for VH-2 versus LH and VH-1 (*p* < 0.0001). The median interval from hysterectomy to first prolapse treatment during the follow-up period was 10 years, with an interquartile range (IQR) of 6–14 years. The median interval from hysterectomy to prolapse surgery was 11 years, with an IQR of 8–14 years.

Fifteen (4%) women received only conservative therapy for their prolapse (pessary or physical therapy). The remaining 51 women (13%) were treated surgically: 10 women from the LH group (7%), 6 from the VH-1 group (6%), and 35 from the VH-2 group (22%). In Table 2 an overview of the procedures performed is displayed. Five women (1%) needed multiple surgeries for prolapse: 1 VH-1 participant underwent anterior colporrhaphy and 4 VH-2 women underwent surgery; 2 anterior colporrhaphy procedures, 1 sacrocolpopexy procedure, and 1 posterior mesh.

**Table 2:** An overview of procedures performed for prolapse after hysterectomy (*N* = 406)

Procedures	LH (*n* = 154)	VH-1 (*n* = 98)	VH-2 (*n* = 158)	Percentage
Anterior colporrhaphy	2	3	7	23.5
Posterior colporrhaphy	4	1	3	15.7
Anterior and posterior colporrhaphy	3	2	1	11.8
Sacrospinous fixation	0	0	8	15.7
Sacrospinous fixation and anterior colporrhaphy	1	0	0	2.0
Sacrospinous fixation and posterior colporrhaphy	0	0	2	3.9
Laparoscopic sacrocolpopexy	0	0	6	11.8
Abdominal sacrocolpopexy	0	0	3	5.9
Vaginal mesh surgery^a^ (3 posterior, 1 anterior, and 1 intravaginal sling)	0	0	5	9.8
Total	10	6	35	100.0

Recurrent procedures are not displayed in this table

*LH* laparoscopic hysterectomy, *VH* vaginal hysterectomy

^a^One posterior mesh procedure and the intravaginal sling were performed for vaginal vault prolapse

### Results of the POP-Q examination

Two-hundred forty-seven women attended the POP-Q examination, of whom 90 were in the LH group (36%), 51 were in the VH-1 group (21%), and 106 were in the VH-2 group (43%). The median time interval between hysterectomy and POP-Q was 16 years, ranging from 13 to 21 years. Table 3 shows an overview of the important outcome variables of this group.

**Table 3:** Outcome Pelvic Organ Prolapse Quantification (POP-Q) examination and questionnaire

Outcome		Total, *N* = 247 (%)	LH, *n* = 90 (%)	VH-1, *n* = 51 (%)	VH-2, *n* = 106 (%)	*p* value	
						LH vs VH-1	VH-2 vs others
POP-Q ≥ stage 2							
Vaginal vault		19 (8)	3 (3)	3 (6)	13 (12)	0.668 *	0.036
Anterior		112 (45)	24 (27)	17 (33)	71 (67)	0.519	<0.0001
Posterior		71 (29)	20 (22)	13 (25)	38 (36)	0.815	0.046
Any compartment		153 (62)	38 (42)	26 (51)	89 (84)	0.408	<0.0001
Prolapse beyond hymen							
Vaginal vault		12 (5)	2 (2)	1 (2)	9 (8)	1.00 *	0.033*
Anterior		29 (12)	4 (4)	6 (12)	19 (18)	0.169 *	0.015
Posterior		14 (6)	3 (3)	1 (2)	10 (9)	1.00 *	0.048*
Any compartment		37 (15)	6 (7)	6 (12)	25 (24)	0.466	0.002
Point C median (IQR)		−5 (−6 to −4)	−5 (−7 to −4)	−5 (−6 to −4)	−4 (−5 to −3)	0.421 †	<0.0001**
POP surgery in follow-up time							
Vaginal vault		13 (5)	1 (1)	0 (0)	12 (11)	1.000 *	<0.0001*
Anterior		13 (5)	4 (4)	2 (4)	7 (7)	1.000 *	0.596
Posterior		11 (4)	5 (6)	1 (2)	5 (5)	0.418 *	1.000*
Any POP surgery		31 (13)	6 (7)	3 (6)	22 (21)	1.000 *	0.001
Bulging symptom, mild, moderate, or severe		32 (13)	8 (9)	5 (10)	19 (18)	1.00	0.068
Symptomatic prolapse, any ≥ stage 2 POP with bulging symptom		27 (11)	5 (6)	4 (8)	18 (17)	0.723*	0.015
Composite outcome, prolapse > hymen or bulging or POP surgery [19]		74 (30)	14 (16)	12 (24)	48 (45)	0.344	<0.0001
Overall PFDI score, median (interquartile range)		37 (17–69)	30 (10–59)	36 (15–60)	46 (23–77)	0.731 †	0.063**

*LH* laparoscopic hysterectomy, *VH* vaginal hysterectomy

*Fisher’s exact test was used to calculate significance instead of Chi-squared test

**Independent *t* samples test was used

In the VH-2 group, all types of POP occurred significantly more often than in the combined LH/VH-1 group. No significant differences were found between the LH women and the VH-1 women at any level. Eighty-four percent of the VH-2 women (*n* = 89) had a ≥ stage 2 prolapse in at least one compartment, versus 42% of the LH women (*n* = 38) and 51% of the VH-1 (*n* = 26) women (*p* < 0.001).

### Combining questionnaire with POP-Q results

In the total POP-Q population, 27 women (11%) had a symptomatic prolapse (defined as bulging plus any ≥ stage 2 prolapse) post-hysterectomy after a median follow-up of 16 years. The results per group are displayed in Table 3.

Thirty percent of our population (*n* = 74) fulfilled the composite outcome (Barber criteria: prolapse beyond the hymen, and/or bulging, and/or a history of POP surgery): 16% of the LH group (*n* = 14), 24% of the VH-1 group (*n* = 12), and 45% of the VH-2 group, *n* = 48 (*p* < 0.0001; Table 3).

Anatomical vaginal vault prolapse ≥ stage 2 was found in 19 women (8%), with or without symptoms. Combining this with women who underwent surgery for apical compartment prolapse in the follow-up period, 31 women in total had a vaginal vault prolapse after hysterectomy; 4.4% (*n* = 4) in the LH group, 5.8% of the VH-1 group (*n* = 3), and 23% in the VH-2 group (*n* = 24). The prevalence of vaginal vault prolapse in our POP-Q population is 13%. An overview per subgroup is presented in Table 4.

**Table 4:** Overview of the prevalence of vaginal vault prolapse

Population	Variables	LH	VH-1	VH-2	Total	*p* value	
						LH vs VH-1	VH-2 vs others
POP-Q population (*n* = 247)	Surgery for vaginal vault prolapse	1	0	12	13		
	Point C ≥ −1 at POP-Q	3	3	13	19		
	Total (%)	4/90 (4.4)	3/51 (5.8)	24^a^/106 (23)	31^a^ (13)	0.707	<0.001
Only questionnaire (*n* = 159)	Surgery for vaginal vault prolapse	0	0	9	9		
Total population (*N* = 406)	Total (%)	4/154 (2.6)	3/94 (3.2)	33^a^/158 (21)	40^a^ (10)	0.785	<0.001

*LH* laparoscopic hysterectomy, *VH* vaginal hysterectomy

^a^One person had a recurrent vault prolapse after apical surgery

### Univariate logistic regression

When analyzing the effect of confounding factors such as age, BMI, and obstetric history, we found no changes in outcome. Using univariate analysis of variance for age and parity (the two variables that were unequal at baseline), the VH-2 as an independent factor still correlated significantly with the presence of a prolapse (F-value = 25.1 with *p* < 0.0001).

### Indication of response bias

Of the women who attended the POP-Q examination, 32 experienced bulging (13%). This was only 5% (*n* = 8 out of 159) for women who filled out the questionnaire but did not attend the POP-Q examination. Of the nonresponders who responded to the telephone interview, 14% experienced bulging symptoms (*n* = 6 out of 42).

## Discussion

### Main findings

In conclusion, the prevalence of symptomatic prolapse (any ≥ stage 2 POP with bulging symptom) 16 years after hysterectomy was 11% in our study population. Vaginal vault prolapse occurred in 13% of the women after hysterectomy: 4.4% after LH, 5.8% after VH-1, and 23% after VH-2. No difference was found in the prevalence of POP between LH and VH for benign indications other than prolapse. POP after VH for prolapse occurs more frequent than after hysterectomy for other benign indications.

Forty-two percent of the LH women had a POP, but only 13% of these women experienced bulging symptoms. These numbers were not statistically significantly different than women with VH performed for nonprolapse indications. However, of those who underwent VH for prolapse, 84% presented with POP at this point in time, with nearly 20% having symptomatic POP, which emphasizes the statement that prolapse is a chronic condition [9, 24].

The most important finding of our study is the similarity of the prevalence of POP in the LH group and the VH-1 group. LH and VH-1 have not been compared before, but a comparison between VH for indications other than prolapse and abdominal hysterectomy showed that VH was associated with a higher risk of POP [11]. The main explanation for this difference was the preoperative descent and space needed to perform VH, assuming a pre-stage of POP in VH patients [8]. As the laparoscopic approach hypothetically seems less traumatic to the uterosacral ligaments than abdominal hysterectomy, we expected to find a larger difference between LH and VH-1. This was not the case, suggesting that the risk of prolapse might not be dependent on the route of hysterectomy. This reiterates the position of VH as the first-choice treatment for benign disease. Thus, the risk for prolapse is not dependent on the route of hysterectomy, but the indication of prolapse for hysterectomy is a major risk factor for recurrence.

### Interpretation

In our literature search, we found one publication [25] showing data of anatomical POP 8 years after laparoscopy-assisted vaginal hysterectomy (LAVH). The frequency of vaginal vault prolapse ≥ stage 2 was low and only slightly different than our study (Shen 1.3% versus our results 3%). The follow-up time of our study was twice as long, which may explain the difference. No data were presented regarding treatment for prolapse or POP symptoms.

Our POP surgery rate in the LH group (7%) was quite similar to a study by Müller et al. [14], who studied the frequency of POP surgery after different types of hysterectomy by questionnaire (follow-up 6 months to 6 years). Following LAVH or LH (*n* = 117), 11 patients (9.4%) needed POP surgery, which seems high considering the short follow-up period.

Vault prolapse after VH for prolapse occurred in 23% of our study population, which is much higher than the previously reported 11.6% by Marchionni et al. [26]. The difference is likely due to the following; the prevalence of vaginal vault prolapse at POP-Q was very similar, but in our study the number of surgeries performed for vaginal vault prolapse was much higher than in Marchionni et al. This may be due to more awareness of pelvic symptoms and an increase in knowledge amongst women about available treatment options compared with 20 years ago. Another contributing factor could be our current knowledge about the importance of the repair of level 1 support at POP surgery, therefore increasing the amount of apical procedures. The high prevalence of vault prolapse after VH-2 supports the statement that hysterectomy without additional surgery should not be considered the first-choice treatment for level 1 prolapse [27].

The overall prolapse surgery rate after hysterectomy for benign indications was 12% according to Lykke et al. [7], which is confirmed by our own rate of 13%.

Previous research described most patients undergoing prolapse surgery within the first 5 years after hysterectomy [8]; however, we did not observe this finding: the median interval from hysterectomy to prolapse surgery was 11 years, with an IQR of 8–14 years. Perhaps this is due to the longer follow-up period in our study or the difference in study design: national registry data versus volunteer participation. The increasing prevalence over time seems logical, as age is also an independent risk factor for POP.

It is important to address the low symptom rate of women with prolapse. Only 32 women experience bothersome bulging symptoms, out of 153 women with a ≥ stage 2 prolapse. When interpreting these data it is important to keep in mind that this is a noncare-seeking cohort. Nevertheless, it would be interesting to follow up on this group and see if symptoms develop over time. It is also worth debating whether bulging is the only relevant symptom that should be taken into account in the definition of “symptomatic prolapse,” as the total PFDI scores were much higher. In the study by Tan et al. [22], 77% of women with ≥ stage 2 POP reported bulging, versus 21% in our population. This is likely due to the difference in setting. Nevertheless, women with prolapse who do not experience bulging, do report significant bother at other PFDI items. It would be interesting to investigate if the current definition of symptomatic prolapse is accurate enough.

### Strengths and limitations

This study shows new data on long-term prolapse after different approaches to and indications for hysterectomy using both a symptom inventory and a POP-Q examination. One of the strengths of this study is that we studied the long-term natural progression of a post-hysterectomy population; therefore, this study gives a realistic image of this patient group. The long follow-up time with high response rate is unique in this area of research. Possible confounders such as obstetric history and age were analyzed and did not influence outcome. Recall bias was minimized by cross-referencing information provided by the participants with their hospital charts.

This study is not without limitations. We attempted to provide an estimation of response bias; however, the information we gathered by phone (pelvic floor complaints and prolapse history) was not sufficient for a good comparison of the responders and nonresponders: the response bias is also affected by confounding factors, which we did not investigate in this group. Owing to our choice of a long follow-up period, loss to follow-up is inevitable and thus our study is subject to follow-up bias. A limitation is the lack of POP-Q data pre-hysterectomy, and therefore not being able to analyze pre-existent uterine descent, especially in the VH-1 group. These factors need to be kept in mind when interpreting our results.

The size of the study population was reliant on the response of the cohort; thus, a sample size calculation was not performed. The population size was small, but we were able to show a very clear difference between VH-2 and the others. The lack of difference between LH and VH-1 makes us wonder if this is due to the small population. However, given the equal prevalence of POP for the VH-1 and LH group, and a significantly higher parity in the VH-1 group, we believe that a significant difference in POP in a larger cohort would come from this confounding factor rather than the intervention.

## Conclusion

Our research contributes to epidemiology regarding post-hysterectomy prolapse and pelvic floor symptoms. For clinicians, this study may improve the counseling of patients undergoing hysterectomy for benign disease regarding their risk of prolapse in the long-term future. In particular, women opting for hysterectomy because of POP should be counseled that the recurrence rate is very high, but the symptom rate is low, and only 21% of them needs additional surgery during the 16 years following the index surgery. Post-hysterectomy patients should ideally be counseled about preventive options such as lifestyle interventions or pelvic floor training.

## References

[1] Jelovsek JE, Maher C, Barber MD (2007). Pelvic organ prolapse. Lancet.

[2] Slieker-ten Hove MC, Pool-Goudzwaard AL, Eijkemans MJ, Steegers-Theunissen RP, Burger CW, Vierhout ME (2009). The prevalence of pelvic organ prolapse symptoms and signs and their relation with bladder and bowel disorders in a general female population. Int Urogynecol J Pelvic Floor Dysfunct.

[3] Mant J, Painter R, Vessey M (1997). Epidemiology of genital prolapse: observations from the Oxford Family Planning Association study. Br J Obstet Gynaecol.

[4] de Boer TA, Slieker-Ten Hove MC, Burger CW, Kluivers KB, Vierhout ME (2011). The prevalence and factors associated with previous surgery for pelvic organ prolapse and/or urinary incontinence in a cross-sectional study in the Netherlands. Eur J Obstet Gynecol Reprod Biol.

[5] Detollenaere RJ, den Boon J, Kluivers KB, Vierhout ME, van Eijndhoven HW (2013). Surgical management of pelvic organ prolapse and uterine descent in the Netherlands. Int Urogynecol J.

[6] Olsen AL, Smith VJ, Bergstrom JO, Colling JC, Clark AL (1997). Epidemiology of surgically managed pelvic organ prolapse and urinary incontinence. Obstet Gynecol.

[7] Lykke R, Blaakaer J, Ottesen B, Gimbel H (2015). Pelvic organ prolapse (POP) surgery among Danish women hysterectomized for benign conditions: age at hysterectomy, age at subsequent POP operation, and risk of POP after hysterectomy. Int Urogynecol J.

[8] Altman D, Falconer C, Cnattingius S, Granath F (2008). Pelvic organ prolapse surgery following hysterectomy on benign indications. Am J Obstet Gynecol.

[9] Dallenbach P, Kaelin-Gambirasio I, Dubuisson JB, Boulvain M (2007). Risk factors for pelvic organ prolapse repair after hysterectomy. Obstet Gynecol.

[10] Blandon RE, Bharucha AE, Melton LJ, Schleck CD, Zinsmeister AR, Gebhart JB (2009). Risk factors for pelvic floor repair after hysterectomy. Obstet Gynecol.

[11] Forsgren C, Lundholm C, Johansson AL, Cnattingius S, Zetterstrom J, Altman D (2012). Vaginal hysterectomy and risk of pelvic organ prolapse and stress urinary incontinence surgery. Int Urogynecol J.

[12] Blandon RE, Bharucha AE, Melton LJ, Schleck CD, Babalola EO, Zinsmeister AR (2007). Incidence of pelvic floor repair after hysterectomy: a population-based cohort study. Am J Obstet Gynecol.

[13] Dutch Central Bureau for Statistics; statline.cbs.nl, the Netherlands. Last updated: 5 February 2014; accessed 6 June 2019.

[14] Müller A, Thiel FC, Renner SP, Winkler M, Haberle L, Beckmann MW (2010). Hysterectomy—a comparison of approaches. Dtsch Arztebl Int.

[15] Schindlbeck C, Klauser K, Dian D, Janni W, Friese K (2008). Comparison of total laparoscopic, vaginal and abdominal hysterectomy. Arch Gynecol Obstet.

[16] Aarts JW, Nieboer TE, Johnson N, Tavender E, Garry R, Mol BW (2015). Surgical approach to hysterectomy for benign gynaecological disease. Cochrane Database Syst Rev.

[17] DeLancey JO (1992). Anatomic aspects of vaginal eversion after hysterectomy. Am J Obstet Gynecol.

[18] Von Elm E, Altman DG, Egger M, Pocock SJ, Gøtzsche PC, Vandenbroucke JP, Initiative STROBE (2007). The strengthening the reporting of observational studies in epidemiology (STROBE) statement: guidelines for reporting observational studies. Lancet.

[19] Barber MD, Brubaker L, Ingrid Nygaard TL, Wheeler II, Schaffer J, Chen Z (2009). Defining success after surgery for pelvic organ prolapse. Obstet Gynecol.

[20] Utomo E, Blok BF, Steensma AB, Korfage IJ (2014). Validation of the pelvic floor distress inventory (PFDI-20) and pelvic floor impact questionnaire (PFIQ-7) in a Dutch population. Int Urogynecol J.

[21] Moalli PA, Jones Ivy S, Meyn LA, Zyczynski HM (2003). Risk factors associated with pelvic floor disorders in women undergoing surgical repair. Obstet Gynecol.

[22] Tan JS, Lukacz ES, Menefee SA, Powell CR, Nager CW, San Diego pelvic floor consortium (2005). Predictive value of prolapse symptoms: a large database study. Int Urogynecol J Pelvic Floor Dysfunct.

[23] Bump RC, Mattiasson A, Bo K, Brubaker LP, DeLancey JO, Klarskov P (1996). The standardization of terminology of female pelvic organ prolapse and pelvic floor dysfunction. Am J Obstet Gynecol.

[24] Moalli PA, Shand SH, Zyczynski HM, Gordy SC, Meyn LA (2005). Remodeling of vaginal connective tissue in patients with prolapse. Obstet Gynecol.

[25] Shen CC, Wu MP, Lu CH, Huang EY, Chang HW, Huang FJ (2003). Short- and long-term clinical results of laparoscopic-assisted vaginal hysterectomy and total abdominal hysterectomy. J Am Assoc Gynecol Laparosc.

[26] Marchionni M, Bracco GL, Checcucci V, Carabaneanu A, Coccia EM, Mecacci F (1999). True incidence of vaginal vault prolapse. Thirteen years of experience. J Reprod Med.

[27] Schulten SFM, Detollenaere RJ, Stekelenburg J, IntHout J, Kluivers KB, van Eijndhoven HWF (2019). Sacrospinous hysteropexy versus vaginal hysterectomy with uterosacral ligament suspension in women with uterine prolapse stage 2 or higher: observational follow-up of a multicentre randomised trial. BMJ.

